# 

**Published:** 1880-06

**Authors:** 


### CONTENTS:

ORIGINAL COMMUNICATIONS:

I. Perinephritis; Fifteen Additional Cases in Children, Completing a Total of Twenty-eight. Remarks on Diagnosis and Prognosis. By V. P. Gibney, M.D ..... 562
II. Diseased Meats and Foul Water, Considered as Causes of Disease. By Henry M. Lyman, M.D. ..... 588
III. Cases of Tracheotomy and Diphtheria. By E. Fletcher Ingals, A.M., M.D. ..... 594

CLINICAL REPORTS:

*Notes from Private Practice.*

IV. A Severe Case of Puerperal Eclampsia at the Eighth Month of Pregnancy, with Recovery. By E. Day, M.D. ..... 597

SOCIETY REPORTS:

V. Illinois State Medical Society ..... 600
VI. Michigan State Board of Health ..... 609
VII. State Microscopical Society of Illinois 613

FOREIGN CORRESPONDENCE:

VIII. Vienna Letter ..... 615

DOMESTIC CORRESPONDENCE:

IX. Examinations of the Illinois State Board of Health ..... 620

REVIEWS AND BOOK NOTICES ..... 621
Books and Pamphlets Received ..... 626
EDITORIAL ..... 627

SELECTIONS:

Two New Anæsthetics ..... 628
Contamination of Drinking Water by Filtration of Organic Matter through the Soil ..... 635

SUMMARY:

*Practical Medicine:*
Deformities of the Vulva Produced by Defloration, Masturbation and Prostitution... 642
Local Morbid Temperature in Affections of the Abdomen ..... 646
A Simple Method of Cure for Ozena ..... 650
Precocity ..... 651
Experimental Study on the Treatment of Hepatic Colic ..... 651
Observations on the Digestion of Milk ..... 652
Cure of Naso-Pharyngeal Polypi by Interstitial Injections of Chloride of Zinc ..... 653

*Surgery:*
Ablation of a Goitre; Cure in Six Days... 654
The Porro Operation ..... 656

*Gynæcology:*
Displacements of the Uterus—A New Theory of their Mechanism and Treatment ..... 659
Diagnosis and Treatment of Obstetric Cases by External Manipulation ..... 661

*Otology and Ophthalmology:*
Communication between the Endo- and Peri-lymphatic Cavities of the Labyrinth and Extra-labyrinthine Intra-cranial Cavities ..... 663
Experiments on the Inoculation of the Cornea, Iris and Conjunctiva with Tubercle ..... 665
On the Disturbance of Vision Caused by Injuries to the Skull ..... 666
Removal of a Foreign Body from the Vitreous Chamber by means of a Magnet ..... 667
On the Sensibility of the Organ of Hearing ..... 668
A Rare Form of Opacity of the Cornea ... 668
On the Chorda Tympani Nerve ..... 669

*Therapeutics:*
The Topical Use of Ergot ..... 670

ITEMS ..... 599, 614, 619, 626, 641
ANNOUNCEMENTS FOR THE MONTH ..... 672
Notice to Correspondents ..... xxii advts.

